# Implementing Standards-Based Grading to improve learning and retention in a meat science course

**DOI:** 10.1093/tas/txag155

**Published:** 2026-10-05

**Authors:** Aeriel D Belk

**Affiliations:** Department of Animal Sciences, Auburn University, Auburn, AL 36832, United States

**Keywords:** alternative grading, meat science, pedagogy, retention, standards-based grading, test anxiety

## Abstract

A growing problem in higher education is the shift of student focus from learning to achieving a specific grade. In an effort to combat this, we applied Standards-Based Grading (SBG) to an upper-level animal sciences course, Muscle Foods. The underlying principle of SBG is that student learning is a process, rather than an endpoint, and as such students should be able to continue academic growth beyond the summative examination. In brief, SBG involves creating defined learning objectives and measuring student progress towards mastering each of them throughout the semester. A key component is that students are able to re-assess on the objectives as their knowledge and understanding improves. In Muscle Foods, we provided 40 learning objectives, which were initially assessed through midterm exams, laboratory activities, and a written project. After receiving scores, students could request a re-test by demonstrating they have applied a new study technique or other method to improve. After three semesters under SBG we found that students received high grades, primarily A’s, and felt more confident in their knowledge of the course. Notably, this method also greatly reduced exam anxiety and student stress. Students indicated that they preferred SBG to traditional grading methods. There are several challenges to implementing the method as well; students also feel that their peers may be “gaming the system” and making scores unfair, it requires significant instructor time investment, and it can be difficult to scale. However, the flexibility of the system allows instructors to adapt it for specific circumstances. We believe this strategy could be applied to great effect in other animal science courses and improve our system of undergraduate education.

## Teaser

Standards-Based Grading, in which students have multiple opportunities to demonstrate mastery of distinct learning objectives, can be a valuable tool to improve student learning, course enjoyment, and anxiety in meat science courses.

## Introduction

It has been noticed by education professionals over the last few decades that the goals of students are shifting from gaining knowledge to just achieving a high grade (Pollio and Beck, 2000; Townsley and Schmid, 2020; Zimmerman, 2020). The questions “will this be on the exam?” and “can you round my score up?” are heard so commonly by faculty that they have become a cliché. For instructors trying to re-focus a course on imparting information rather than grades, one tool is modifying the assessment system itself. In an upper-level animal science course, Muscle Foods, Standard-Based Grading (SBG) was implemented as a method to shift students’ perceptions of the class purpose. The objectives of this study were to (1) demonstrate to what extent the SBG method improved student learning and understanding and (2) evaluate the impact of SBG on student stress and exam anxiety. This study found that grades and student knowledge retention were high under SBG, and, perhaps more importantly, that SBG improved student mental loads and increased their overall enjoyment of the subject. This may be a method that other courses within animal science curricula would also benefit from alternative grading methods, therefore this study also presents the steps and considerations used to implement this system.

## Introduction to Standards-Based Grading

There are many alternative grading systems used in college-level science education programs, including mastery grading, specification grading, and ungrading; but, SBG is the most common (Hackerson et al. 2024). These are all labeled “alternative” in relation to traditional or conventional grading, in which points are given for correct answers and the percentage of rewarded points is the final grade. SBG addresses inherent issues in this by instead rewarding students understanding of topics measured through defined learning objectives (LO). Standards-based grading was selected as the course was already structured with objectives, and that made it a ready fit for altering the course.

Standards-Based Grading can be defined through four core principles, as synthesized by Townsley and Wilcox (2024): (1) grading communicates students’ current learning based on a standard; (2) a finite number of performance categories are used to assess learning; (3) grades are assigned by summative assessments rather than formative; (4) students have multiple opportunities to demonstrate their learning. In practice, this is generally conducted by first identifying standards, commonly included as Learning Objectives (LO). These should be distinct, measurable goals that can be assessed against a proficiency scale or rubric. In the early iterations of SBG, each LO is either passed or failed, but more recently it became common to rank performance on a 3 to 5 point scale, demonstrating progress towards mastery (Link and Guskey 2022). Then, critically, students must have opportunities to re-assess their mastery on each objective. This can be done through new examinations or quizzes or project options; however, in large courses this can also be administered through tiered assessment levels, using a quiz, then unit exam, then final exam (Zimmerman 2020). An optional but useful element of this is requiring students to evaluate their studying strategies before being allowed to repeat assessments. Overall, this educational approach has been found to improve metacognitive skills as students reflect on their learning strategies, improved grades, and improved student comfort (Boesdorfer et al. 2018).

## Methods for course development

### Course description

The introductory meat science course, titled Muscle Foods, is a 4-credit course offered primarily to upper-level undergraduate animal science students at Auburn University (Auburn, AL). It was available as a directed elective until 2024 when a curriculum change converted it to a core requirement for animal science majors. It is structured with three 50-minute lectures and one 2-hour lab section each week; the lectures are led by faculty and lab sections led primarily by graduate student teaching assistants (TA) with support from the faculty and meat lab staff. Prior to the introduction of the assessment method discussed here, student learning was assessed through four online exams using a course management tool, a short oral presentation, and a written assignment. The overall description and objectives, as defined by the course catalog, are “introduction to domestic animal growth and development, comparative evaluation of livestock and carcass composition.” Biochemical and physiological factors affecting fresh meat and processed meat quality. Fundamentals of ante-and postmortem muscle biology, slaughter, processing, storage and merchandising of meat and meat products. Notably, due to the curriculum structure, students often take this course alongside other classes considered difficult, including animal reproduction, organic chemistry, and microbiology. This study was conducted over three fall semesters during which SBG was applied. The total student enrollment was 122, with the three semesters consisting of 31, 41, and 50 students. To ensure anonymity and compliance with the Family Educational Rights and Privacy Act, no demographic information was collected on the students.

#### Selection of learning objectives

Creating defined and distinct learning objectives (LOs) is a fundamental component of the SBG system, therefore generating these was the first step in redesigning the course structure. The overall objectives of the course were already defined, so the LOs were developed within this framework using Bloom’s Taxonomy guidelines (Adhikari 2024; Townsley and Wilcox 2024). First, the specific knowledge a student should have upon completing the course was determined. Then, a total number of LOs for the course was set. The final step was to write a testable objective representing the important topics, limiting the number to something manageable for the 15-week course. Upon implementation, the LOs were revised each semester to make sure they are each distinct and equal in the amount of class knowledge they encompassed. This publication will present the finalized versions of the LOs finalized after three semesters using this methodology.

Course topics were generated from the overall course objectives and one topic was assigned per week (Table 1). The goal was to include 2–3 LOs per topic, resulting in approximately 30 total learning objectives. When the actual course material was evaluated, these numbers were adjusted slightly to maintain specificity and even size among the LOs. For example, initially the topic “properties of fresh meat” was divided into two LOs: “Describe the factors that influence meat quality” and “identify meat quality defects.” But, the first LO was complex and difficult to assess, so it was further divided to have LOs addressing color and water holding capacity separately. A list of example questions used to assess the lecture objectives can be found in Table 2. The lab section of the course also needed to be addressed in the LOs, primarily to justify student time and effort during these activities. It was learned through experience that students will not put effort into an activity that is not directly tied to their grade, unfortunately. It was decided that five LOs would appropriately demonstrate understanding of lab activities. Similarly, four LOs were assigned to other course assignments (oral presentation, written project). All LOs were weighted equally for the purposes of final grade determination. The full course syllabus, defining the LOs and containing the methods by which SBG was communicate to the students, is available in the supplementary materials, available as supplementary data at *Translational Animal Science* online.

**Table 1:** Learning objectives used in the Muscle Foods course for Standards-Based Grading evaluation and the method of preliminary assessment.

Course topic	Learning objective	Assessment
**Meat as food**	Describe the role of meat in the U.S. and global cultures and economies	Exam 1
**Structure, composition, development of animal tissues**	Illustrate and label the key structural components and myofilaments that make up muscle tissue	
	Compare the different muscle fiber types and fatty acids within the livestock animal	
	Describe the process of muscle formation and growth	
**Muscle contraction and metabolism**	Describe the process of muscle contraction and relaxation	
	Discuss the metabolic processes for generating energy for muscle contraction and how these can influence meat quality	
**Conversion of muscle to meat**	Outline the processes that occur in the conversion of muscle to meat	
**Meat inspection and food safety**	Explain the role of meat inspection in the production of safe food	Exam 2
	Understand the basics of HACCP and preventative food safety and identify potential hazards	
**Meat grading and evaluation**	Describe the purpose and methods for assessing meat quality and composition	
	Calculate yield and quality grades for beef, pork, and lamb	
**Meat merchandising**	Compare the pros and cons of different meat merchandising methods	
**Animal handling**	Identify proper and improper animal handling conditions and potential impacts on meat products	
**Properties of fresh meat**	Describe the factors that influence water holding capacity in meat	Exam 3
	Describe the factors that influence meat color	
	Identify meat quality defects and recognize the causes	
**Packaging and storage of meat**	Diagram the four primary drivers of meat spoilage	
	Diagram the process of lipid oxidation	
	Compare the advantages and disadvantages of different packaging methods	
	Describe refrigeration, freezing, and drying practices for meat preservation	
**Principles of meat processing**	Classify the different types of processed meat products	
	Describe the raw materials and ingredients used in meat processing	
	Describe the meat cure reaction	
**Microbiology of meat**	Recognize potential sources and routes of microbial contamination in meat and food products	Exam 4
	Explain the factors that make meat a good microbial growth medium	
	Classify and categorize the primary foodborne microorganisms	
**Palatability and cookery**	Identify the key factors that are involved in meat palatability and consumer acceptance	
	Understand the events that occur during cooking meat products	
**Nutritive value**	Discuss the value of meat in the diet	
**By-products and food waste**	Identify the uses for edible and inedible meat byproducts	
	Describe the issue of food waste and methods that can be used to reduce it	
**Lab section**	Demonstrate the correct use of meat lab PPE and knife safety	Lab final, participation
	Describe the steps of animal harvest	
	Outline the process of meat fabrication, from carcass to retail cuts	
	Identify meat processing equipment and meat retail cuts	
	Actively participate in hands-on and laboratory activities	
**Current issues**	Debate current issues in meat and animal sciences	Presentation
**Applications of course knowledge to industry**	Use class knowledge to design a practical and realistic meat processing facility	Written assignment
	Demonstrate awareness of meat safety and quality considerations in practice	
	Demonstrate awareness of meat safety and quality considerations in practice	

**Table 2:** Example questions demonstrating the type of assessments used for learning objectives during the semester.

Learning objective	Example questions
**Outline the processes that occur in the conversion of muscle to meat**	Label the 4 phases of rigor mortis on a graph. Describe the process of normal pH decline that occurs postmortem. Include the compounds that drive pH drop and the expected pH values. In as much detail as possible, describe the processes that lead to the PSE condition in pork. Include at least one genetic cause and how it impacts the biochemical processes that occur postmortem.
**Compare the pros and cons of different meat merchandising methods**	Assign each species the appropriate IMPS series: Beef, Lamb, Pork, Goat. Describe one specific supply chain by which meat goes from the harvest facility to the consumer. You should describe at least three locations in this answer. Why do we cut primals into subprimals? Use the principles of meat cutting to guide your answer. Compare and contrast the advantages and disadvantages of the three main packaging types: tray overwrap, vacuum, MAP. Specifically reference shelf-life, storage and transportation ease, and consumer acceptance.
**Describe the factors that influence water holding capacity in meat**	Where is most muscle water located? What are the three forms of biological water? Provide the name and a short description of what this means. You are approached by a producer that is having a problem with some of their fresh beef products. After fabrication, the product is losing more water from purge than normal, and consumers have complained that the product is tough and has a strange texture. What is one problem that may be occurring in their process? Why would that problem result in these quality issues? What further questions or investigation would you use to confirm your guess?
**Recognize potential sources and routes of microbial contamination in meat and food products**	Which of the microbes discussed in class are spread through a fecal/oral route? Describe three potential locations during beef harvest and fabrication where microbial contamination of the carcass/product could occur. Why are these high risk contamination activities? This picture went viral last year as an example of improper food handling. As a meat scientist, you feel you should correct the error. Please write a comment or tweet-style response to this post that would help educate the public about why this is incorrect and what you should do instead.

Examples were chosen to represent all four course exams.

#### Creation of grading scheme

Though an ideal situation would be to present the outcomes of the course solely through the LOs, the university requires reporting letter grades. Thus, it was necessary to convert the grading scheme back to a traditional score. A 4-point rubric (0 to 3) for each LO was selected based on published resources (Zimmerman 2020). Originally, a rubric was created for each LO outlining what specific knowledge showed each level of mastery (supp material). However, after the first semester of implementation it was determined that students did not use this resource and it ultimately made grading the assessments cumbersome, so a simple, universal rubric was applied after the first semester under the new scheme. Under this, a 0 will be assigned if there is clearly no effort made toward the LO, 1 will be assigned if students have attempted to demonstrate knowledge, a 2 will be assigned if students have shown progress towards mastery or partial mastery, and the 3 will be assigned when students fully demonstrate mastery. With 40 LOs worth up to 3 mastery points each, the course was worth a total of 120 points. To determine the final letter grades, the course was first treated this as traditional scoring system (ie 108 points = 90% = A) but adjusted slightly down to broaden the letter grade categories. This broadening was included to reward student effort with higher grades, though a more scientific approach can perhaps be used by others applying this method with more historical grade distribution data available. These were originally written to be easy cutoffs (ie 100 points for an A), but this was changed in 2025 to increase the rigor and make the grades feel more “earned” following student feedback. The grading scheme is displayed in Table 3.

**Table 3:** Learning objective (LO) points required to achieve each letter grade.

Letter grade	LO points		
	2023	2024	2025
**A**	100–120	100–120	102–120
**B**	85–100	85–100	84–101
**C**	70–85	70–85	66–83
**D**	50–70	50–70	50–65
**F**	< 50	< 50	< 50

Levels were determined by first evaluating the levels as they would be in a traditional grading system (ie 108 points = 90% = A) but adjusted slightly down to broaden the letter grade categories using instructor judgement.

#### Assessments

The use of SBG requires specific assessment to interpret student mastery. There have been many strategies proposed for this, but all generally agree that there needs to be an initial assessment followed by opportunities for repeated re-assessment until students can demonstrate mastery (Zimmerman 2020). For this course, the LOs were assessed initially through one of four methods: in-class exam (31/40, 77.5%), lab participation (5/40, 12.5%), oral presentation (1/40, 2.5%), or written assignment (3/40, 7.5%; see Table 1).

In-class exams were traditional paper exams, divided into sections based on LO. Question types included fill-in-the-blank, matching, short answer, and essay-style questions, with few or no multiple-choice questions as these do not represent overall understanding well. Students are encouraged to make an effort to answer all questions regardless of whether they had completely mastered the LO. Grading was completed by the instructor and TAs. After grades were returned, students had the opportunity to request a re-assessment on up to three LOs per week by submitting a request that highlighted specifically what additional work they have completed to expect better mastery. Students submitted the request on Monday and had one week to complete an online quiz covering that specific LO. Multiple online quizzes for each learning objective were created before the start of the semester to align with the LOs and overall course objectives. Students were able to make a request every week of the semester after the initial assessment for a LO. This re-assessment was graded, and the mastery scored was updated to reflect if they improved. The final exam, at the end of the semester following the university schedule, offers one final re-assessment for every exam-based LO (LOs 1–31).

Lab section LOs (32–36) were assessed using slightly different methods. Students were required to complete a worksheet during lab each week reflective of the weekly topic and the TAs monitored the students for participation. The final LO score was assigned following a group lab final that consisted of practical questions and activities. The oral presentation, a 5-minute presentation and discussion on a surprise current issue in meat sciences, was graded based on a performance rubric and students had the opportunity to repeat their presentation with another topic if they were unsatisfied with their score. Finally, the written presentation was graded according to a rubric based on content, inclusion of course material, and writing ability. Students had one opportunity to make edits and re-submit their written assignment after grades were returned. Further explanations of assignments, rubrics, and grade policies are available in the course rubric (supplemental material, available as supplementary data at *Translational Animal Science* online).

#### Implementation

This novel grading system was explained in detail in the course syllabus (see supplementary material, available as supplementary data at *Translational Animal Science* online) and was specifically introduced to students on the first day of class. The goals of using SBG and how it is expected to influence student outcomes, instructions for completing assessments, and examples of the process were emphasized. The system was reiterated following the first exam, when students were presented with the first opportunity to request re-assessments. Students had free opportunity to ask questions and for clarification throughout the semester.

## Methods for assessing student outcomes

### Survey methods

The usefulness of the SBG method to student learning and retention was assessed through measuring student grades and anonymous surveys. These methods were approved as exempt by Auburn University Institutional Review Board. At the completion of the semester, following the last regular exam, students were administered the survey by a TA to maintain anonymity to the faculty. Students were informed that the survey was optional, and a total of 106 surveys were returned. Survey questions included six Likert-scale questions about student feelings regarding the SBG system and two open-ended questions allowing for more specific feedback (supp material). The Likert-scale questions asked students to respond to six statements on a scale from 1 (strongly disagree) to 5 (strongly agree): (1) The grading system and course expectations were adequately described; (2) This grading system improved my learning and retention of the course material; (3) I expect to receive a higher grade in this course than I would have in traditional grading; (4) I felt more confident going into exams under this system; (5) I felt less anxious going into exams under this system; and (6) I received sufficient feedback in this course. The two free-response questions were: (1) Do you want to provide further explanation of any of your answers above? And (2) Please share any other thoughts, comments, or suggestions about the grading system used in this course. Grades, including number of mastery points and letter grades, were recorded at the end of the semester.

Data were analyzed using R version 4.5.2 (R Core Team, 2013). Grades were analyzed using the Chi Square test. Likert-scale questions were analyzed by comparing differences among semesters using the Kruskal-Wallis test, as recommended by Eiselen and van Huyssteen (2023). Open-ended questions were evaluated first using qualitative data analysis by categorizing the results into common responses and counting the comments that fell under each, then by word choice using the tidyR and tidytext packages (Silge and Robinson 2016; Wickham et al. 2026). Differences were considered significant at *P* < 0.05. All visualizations were constructed using ggplot2 (Wickham 2009).

## Results and discussion

### Standards-based grading resulted in high student grades

Students earned overall high grades by utilizing the re-assessment system in this course. In the three semesters studied, 87.1%, 82.9%, and 96% of students earned an A based on the grade cutoffs for the given semester (Fig. 1). There was no difference (*P* = 0.38) in the overall grade proportions between semesters. In this study, student grades were the only true objective measure for gauging student understanding. As students had the option to re-assess on each LO until they were satisfied with their grade, this is not a perfect metric. Previous work, however, has more concretely demonstrated the improvement in student learning. In teaching mathematics, Zimmerman (2020) found that students taught under SBG scored higher on standardized placement scores after the class than those in other systems and performed better in subsequent courses. Boesdorfer et al. (2018), applying SBG to a non-major chemistry course, showed that students were generally more successful on the second attempt at assessment for a learning objective, indicating that the re-assessment process allowed for iterative improvement. Taken together with the current work, a picture of improved student understanding comes together. Interestingly, in a high school core curriculum, Proulx et al. (2012) found that SBG resulted in a decrease in A and F grades, and an increase in B and C grades. They postulate that this is due to the assessment of higher-level concepts in the assessments and the loss of extra credit opportunities students previously relied on for boosting grades. Compared to the college-level course evaluated here, these challenges are less applicable as expectations are generally higher for collegiate students even under traditional grading. To truly determine the impact of SBG on student learning and knowledge retention, follow-up assessments of students in this course and traditionally graded courses in the years after class completion would be conducted, but this is not currently feasible. Still, high grades coupled with the student feedback described below, suggests SBG has a positive impact on student learning.

**Figure 1:**
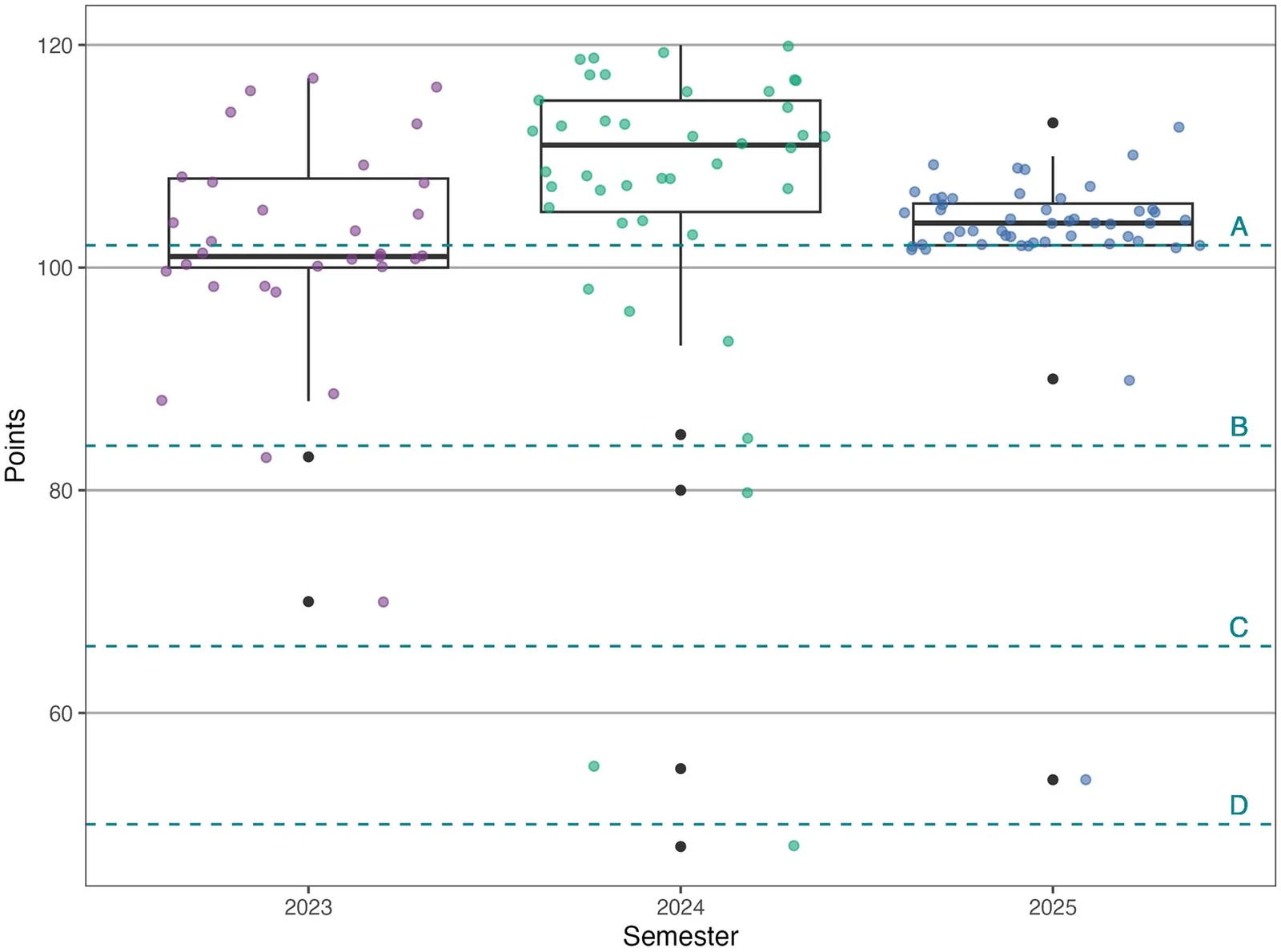
Number of points students earned at the end of the semester. Students could earn up to 3 mastery points for each learning objective, resulting in a maximum possible grade of 120 points. Dashed lines indicate associated letter grade break points.

### Standards-based grading improved student confidence

The subjective impact on students was also assessed using a voluntary end-of-semester survey. Students generally indicated positive feelings regarding the SBG system. There were no differences (*P* > 0.05) among semesters for any Likert-scale question, and overall scores were very high (Fig. 2). The mean scores out of 5 were 4.88, 4.82, 4.64, 4.81, 4.71, and 4.87. The lowest-scoring question, question 3, asked whether students did better than they would have otherwise expected, and it appears that the students indicating a 1 did so primarily because they were confident they would achieve a high grade either way, with one commenting “I kinda already knew the material” in open-ended questions.

**Figure 2:**
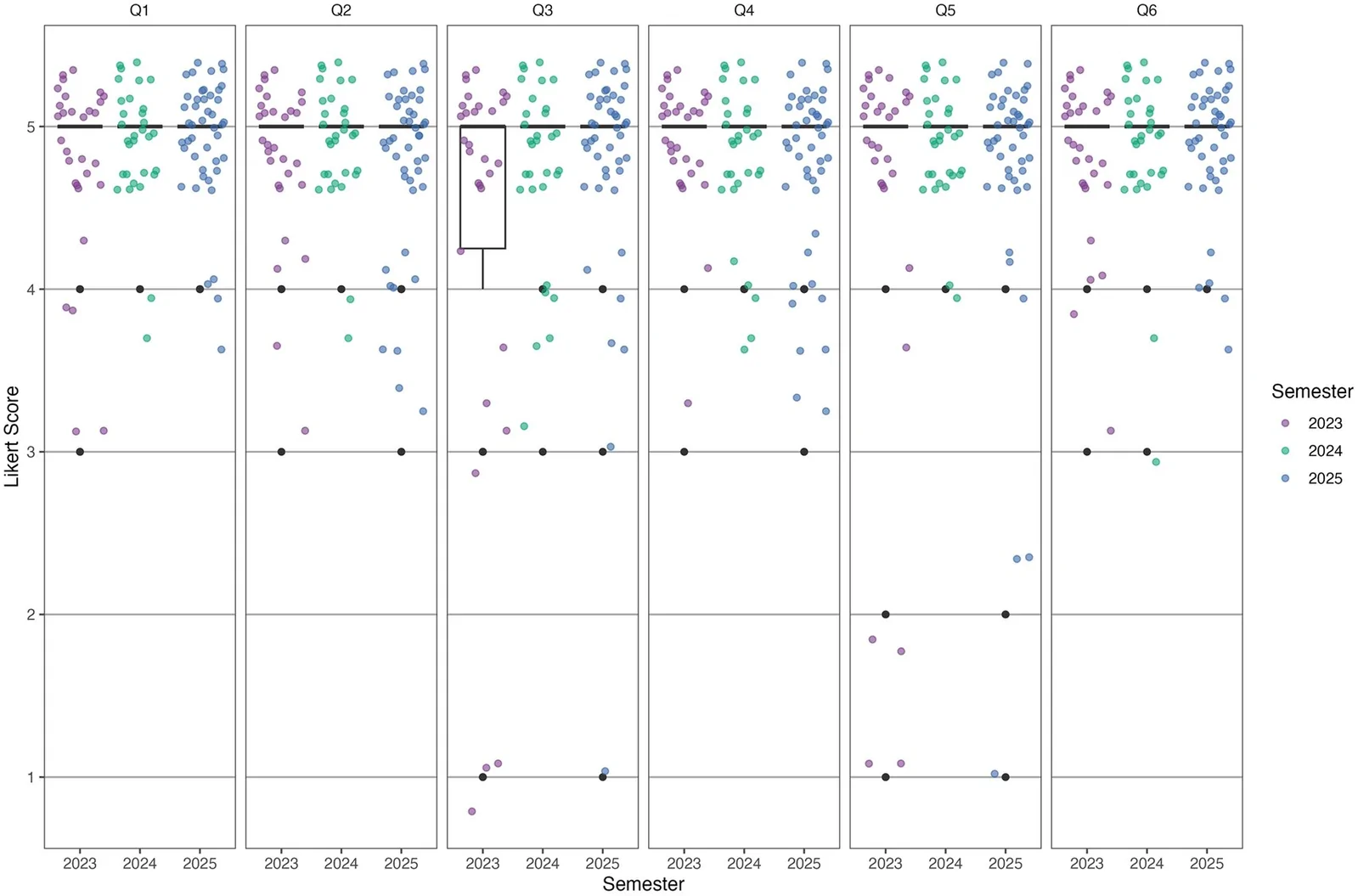
Student responses to six Likert-style questions related to their experience in the course under Standards-Based Grading. The questions were, on a scale of one to five, with five indicating strong agreement: (1) The grading system and course expectations were adequately described; (2) This grading system improved my learning and retention of the course material; (3) I expect to receive a higher grade in this course than I would have in traditional grading; (4) I felt more confident going into exams under this system; (5) I felt less anxious going into exams under this system; and (6) I received sufficient feedback in this course.

The open-ended questions provided more feedback related to what specific aspects students appreciated or were concerned by in the grading system. Overall, 125 comments were submitted that were categorized into 153 general areas following quantitative analysis (Table 4). Students expressed a general appreciation for the course itself (17 comments) and for the grading system (36 comments). Beyond this, comments described a reduction in stress and anxiety (49 comments), improvement in learning and retention (21), positive changes to study habits (10), and that SBG in Muscle Foods improved performance and stress in other classes (7). Students also provided concerns related to the course, but at much lower levels, including concern over other students abusing the system (4 comments), the system being confusing (4), and grading time being too long or inconsistent (3). The concerns regarding confusion and grading were primarily identified in the first semester the system was implemented, and were addressed with modifications in future semesters, as described in the implementation methods above. When analyzing the most commonly used words in the open-ended comments, it was clear that students were focused primarily on exam stress, studying, and knowledge (Fig. 3). The word “loved” was the fifth most frequently used word in these responses. In our opinion, the most notable feedback from students was that the use of SBG in Muscle Foods also helped them improve in other classes because it helped them prioritize studying and reduced stress. One illuminating comment was “it reduced a lot of stress, and it made my performance in other classes much better,” which exactly captures the goals of utilizing this grading system.

**Figure 3:**
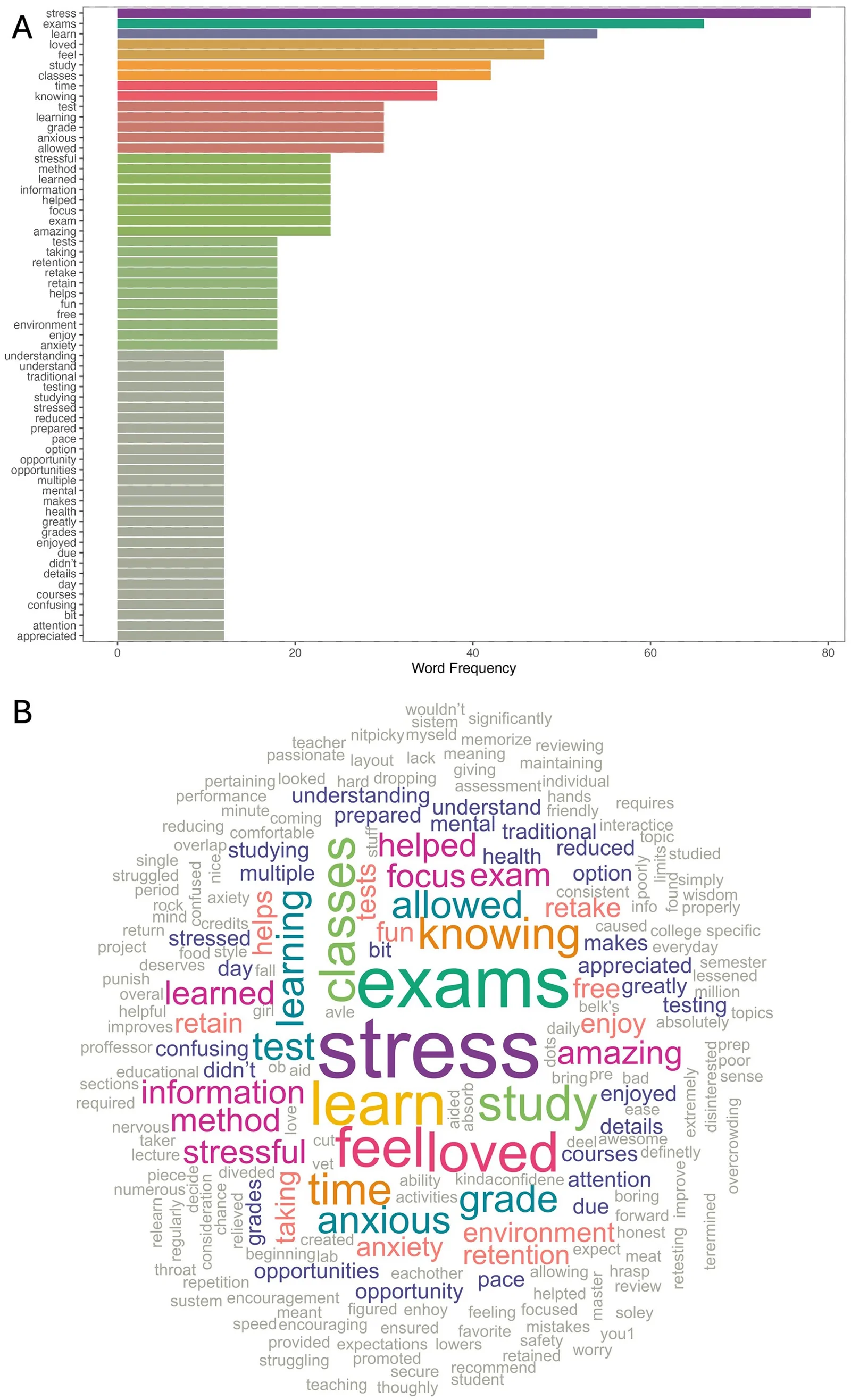
Word frequency analysis of student responses to open-ended questions. The two questions were: (1) Do you want to provide further explanation of any of your answers above? And (2) please share any other thoughts, comments, or suggestions about the grading system used in this course. (A) ranking of the most frequently used words in responses. (B) Word cloud summarizing commonly used words.

**Table 4:** Qualitative analysis of results from the open-ended survey questions provided to students at the end of the Muscle Foods course when Standards-Based Grading (SBG) was used.

Comment category	Number of comments	Example comment
**Generally enjoyed class**	17	Such an amazing teacher and makes class interesting. Lecture is known to be boring in other classes but this class I looked forward to it. This class was an amazing experience, the lab portion opened my eyes to a whole other profession I greatly enjoyed! So much fun!
**Generally liked SBG**	36	I like this system much more than traditional grading. This grading system was by far the best one that I’ve learned under.
**Reduced anxiety and stress**	49	The less anxiety aspect is crucial especially since I’m a panic exam taker, it put less pressure so I felt I did better. I greatly appreciated the opportunity to retest. It improves my grade and extremely lessened my test anxiety.
**Improved student learning/retention**	21	I felt that I actually learned and didn’t just memorize things for the test. I feel like the grading was a bit confusing at first but once I figured it out I really liked it. I like that it doesn’t punish for not knowing but instead makes you study to understand it better. While I do poorly on exams, I feel I have learned in class more than any other class. I get to restudy and figure out my expectation on what I really need to know.
**Improved study habits**	10	I thoroughly enjoyed the feeling that even if I did poorly I could retest. I allowed me to focus on what I already knew while studying and not stress about what I didn’t because I could retest later after reviewing. This system helped me understand what needed improvement.
**Helped with performance in other courses**	7	I was able to focus more on classes that I was struggling in and bring most of those grades up while maintaining a good grade in this class. I really appreciated this system as I was taking 17 credits so this allowed me to learn in my own time.
**Concerns of unfairness or abuse by other students**	4	I liked it, I do think there are ways to abuse it… While I think it’s awesome, it definitely gets taken advantage of. Maybe tighter restrictions on retests.
**Confusion about SBG system**	4	I feel like the grading was a bit confusing at first but once I figured it out I really liked it. some details of the grading were confusing ie learning objective pertaining to meat project and lab activities.
**Grading or re-grading taking too long**	3	The turn around time for submitting retest was great but got kinda iffy at end of semester with crunch time.

Comments were coded into the common themes, and representative example comments were selected for illustration. 125 total comments represented 153 coded responses as some comments fit multiple areas. Example comments were corrected for spelling and shortened to provide the relevant portion for the category.

The broad support of the SBG system by the 122 students included in this study aligns with trends from previous research. Tripp (2018) demonstrated that, in agricultural education, a SBG system led to greater student confidence, and that students and parents appreciated the improved feedback. Chemistry students in the Boesdorfer et al. (2018) study shared the general sentiments found in the current work, including the benefits of completing LOs at one’s own pace. The support for SBG was slightly lower in the chemistry course, however, with 21.6% of students disagreeing with the statement “I like the assessment method in the course.” This can be compared with the approval of students in the current work, in which only 4% of scores were neutral or negative across the six Likert questions. It may be that a course like Muscle Foods has more straightforward skills being assessed compared with a large chemistry course, making it easier for students to adapt to a new system. Further, Boesdorfer et al. (2018) implemented the SBG system such that there was more out-of-class work for the students, which may have also contributed to more negative opinions.

Students did indicate some concerns with SBG, especially in the ability to “game the system” or for some students to take advantage of the leniency. This is difficult to solve without casting potentially biased judgements on students. However, in response to this feedback, potential amendments to the course design for the future include increasing the number of LO mastery points required to achieve an “A” or requiring more justification from students as to why they are prepared to re-assess. Another major impediment to implementing SBG is that it greatly increases the time required by the instructors of the course, which would be especially detrimental if an instructor is leading multiple courses in the same semester. Through the three semesters included in this study, some of these challenges were remediated by engaging teaching assistants in the grading of re-assessments, but this challenge is likely to persisted to some extent, given the necessity of intense grading associated with the method. A related concern is that students may be cheating during the online re-assessments. In an idealized version of SBG implementation these would be proctored, but this is difficult given the time limitations of many instructors. This could be mitigated using educational technologies that monitor student activity and computer use. Finally, SBG, being a very individualized grading system, is difficult to scale to larger courses, but this is possible. In science and engineering courses, this has been accomplished by using multiple grading levels, for example, assignments, quizzes, and exams, allowing students to demonstrate understanding of the same LO multiple times in a more manageable method (Boesdorfer et al. 2018; Zimmerman 2020). This could also potentially be accomplished using peer grading methods. Overall, SBG is challenging to implement, but is flexible enough to adapt to the specific needs of a course.

## Limitations and conclusions

### Limitations

While this study demonstrates the value of the SBG system applied in a meat science course, there are some limitations to the findings presented here. As the instructor only taught one lecture section of the course during one semester a year, there was not a comparable control group to distinctly show improvement in understanding compared with traditional grading beyond the qualitative student views. Additionally, the number of re-assessments taken among students and for each LO were not tracked, which restricted interpretation and analysis here. Another limitation to this study is an inability to demonstrate knowledge retention beyond the course. Future studies in this area could consider using the final examination as a test of knowledge retention rather than an optional re-assessment to mitigate this.

### Conclusions

The primary goals of implementing this grading system were to improve student understanding of course material and reduce student anxieties surrounding testing and assessment. This study demonstrates that, to some extent, both objectives were achieved. Student grades were high, and students responses were overwhelmingly positive each semester the system was utilized. Overall, SBG in meat science courses is one tool that can be used to engage undergraduate students and focus on knowledge attainment rather than learning for the test. Application of SBG requires more effort on the part of the instructor and teaching assistants, but this is a negligible downside compared with the overall benefits to students.

## Supplementary Material

txag155_Supplementary_Data

## List of abbreviations

LO: Learning Objective
SBG: Standards-based grading
TA: Teaching Assistant
