# Supplementary material for: Implementing Standards-Based Grading to improve learning and retention in a meat science course
Source: Transl Anim Sci. 2026 Oct 5;10:txag155. doi: 10.1093/tas/txag155 (PMC13690418; doi:10.1093/tas/txag155)
Supplement: Supplementary file 1 [file txag155_supplementary_data.docx]

**Supplementary Material 1**

Course Syllabus

**ANSC 3700**

**Muscle Foods**

Fall 2025

Introduction to domestic animal growth and development, comparative evaluation of livestock and carcass composition. Biochemical and physiological factors affecting fresh meat and processed meat quality. Fundamentals of ante- and postmortem muscle biology, slaughter, processing, storage and merchandising of meat and meat products.

**Welcome!**

As we prepare to embark on this course related to Meat Science and the conversion of animals to edible products, I hope that you are excited to enhance your knowledge of the meat and food industries. It is my intent that upon completion of this course you will have a greater appreciation of the meat sciences that will aid you in completing additional course work within the Department of Animal Sciences. I am also excited to share the career opportunities that exist within the meat and food industries. My favorite part of meat science is how it touches every field – we will talk about history, biology, chemistry, biochemistry, microbiology, economics, business, and engineering. It is so fun to see all of the different areas come together to help us understand something that is so commonplace! I am here to help you see this, and hopefully pass at least a little of my passion for meat science on to you. Though not all of you may love every aspect of this class, my hope is that each of you will learn at least one new thing that you find exciting and novel that will influence your decisions going forward.

**Course Details**

**The Teaching Team**

INSTRUCTOR:	**Dr. Aeriel Belk**

OFFICE:	Upchurch Hall 232

EMAIL:	aeriel.belk@auburn.edu

PHONE:	(334) 844-1505

TEACHING ASSISTANTS:

**Isabella Gafhana**

igg0009@auburn.edu

Office: Upchurch 140

**Isabella Tolleson**

izt0008@auburn.edu

Office: Upchurch 140

**Noel Claudio-Soto**

nac0037@auburn.edu

Office: Upchurch 140

**Jonah Dunn**

jkd0042@auburn.edu

Learning Assistant

MEAT LAB TEAM:

**Mr. Barney Wilborn**

Meat Lab Manager

wilbobs@auburn.edu

**Erik Hallmark**

Meat Lab Butcher

OFFICE HOURS:

Dr. Belk: by appointment

Teaching Assistants: Wednesdays 12-2pm

Upchurch 238

CONTACT US: The best method to contact your instructors outside of class or office hours is email. When emailing about this course, please include “ANSC3700” in your subject line. Please allow 1-2 days for a response. If you need a more rapid response, please include “urgent” in the subject line, or you can call Dr. Belk’s office phone. You can also send messages in Canvas, but these are not checked as frequently by your instructors, email will result in a quicker response.

- Scheduled Meeting Times
  - **Lecture**
    - Sciences Center Classroom Bldg 118
    - MWF 11:00 - 11:50
  - **Lab**
    - Lambert-Powell Meat Laboratory
    - Section 1: Th 11:00am - 12:50pm
    - Section 2: Th 1:00pm – 2:50pm
- Credit Hours: 4
- Prerequisite: Introduction to Animal Sciences
- Textbook:
  -
**Recommended**: material taught in the course is sourced from the textbook, but there are not assigned readings or questions from only the textbook that is not also included in the lecture
  - **Principles of Meat Science**, Fifth Ed., E.D. Aberle, J.C. Forrest, D.E. Gerrard, E.W. Mills. ISBN13: 9780787247201
- **Laboratory Materials:** Each student is required to purchase supplies for the lab. These can be purchased through the Meat Laboratory for **$100** paid in the form of cash, card, or check payable to Auburn University Meat Laboratory. Purchases will be coordinated the first week of class. Supplies will be stored in meat lab lockers during the semester and removed at the end of the semester. Materials include:
  - Boots
  - Disposable Frocks
  - Disposable Gloves
  - Hair Nets
  - Knives (boning and skinning)
  - Plastic Scabbard
  - Plastic Arm Guard

**Outcomes and Objectives**

**Course Learning Objectives:** This course should provide students with a broad scientific view of the principles of meat science and the meat industry. The goal of this course is to produce students that understand the scientific principles behind decisions made in the meat industry and can discuss current issues in animal agriculture with strong scientific arguments. To achieve this, this course will be taught based on the following **40 learning objectives**:

1. Describe the role of meat in the US and global cultures and economies
2. Illustrate and label the key structural components and myofilaments that make up muscle tissue
3. Compare the different muscle fiber types and fatty acids within the livestock animal
4. Describe the process of muscle formation and growth
5. Describe the process of muscle contraction and relaxation
6. Discuss the metabolic processes for generating energy for muscle contraction and how these can influence meat quality
7. Outline the processes that occur in the conversion of muscle to meat
8. Explain the role of meat inspection in the production of safe food
9. Understand the basics of HACCP and preventative food safety and identify potential hazards
10. Describe the purpose and methods for assessing meat quality and composition
11. Calculate yield and quality grades for beef, pork, and lamb
12. Compare the pros and cons of different meat merchandising methods
13. Identify proper and improper animal handling conditions and potential impacts on meat products
14. Describe the factors that influence water holding capacity in meat
15. Describe the factors that influence meat color
16. Identify meat quality defects and recognize the causes
17. Diagram the four primary drivers of meat spoilage
18. Diagram the process of lipid oxidation
19. Compare the advantages and disadvantages of different packaging methods
20. Describe refrigeration, freezing, and drying practices for meat preservation
21. Classify the different types of processed meat products
22. Describe the raw materials and ingredients used in meat processing
23. Describe the meat cure reaction
24. Recognize potential sources and routes of microbial contamination in meat and food products
25. Explain the factors that make meat a good microbial growth medium
26. Classify and categorize the primary foodborne microorganisms
27. Identify the key factors that are involved in meat palatability and consumer acceptance
28. Understand the events that occur during cooking meat products
29. Discuss the value of meat in the diet
30. Identify the uses for edible and inedible meat byproducts
31. Describe the issue of food waste and methods that can be used to reduce it
32. Demonstrate the correct use of meat lab PPE and knife safety
33. Describe the steps of animal harvest
34. Outline the process of meat fabrication, from carcass to retail cuts
35. Identify meat processing equipment and meat retail cuts
36. Actively participate in hands-on and laboratory activities
37. Debate current issues in meat and animal sciences
38. Use class knowledge to design a practical and realistic meat processing facility
39. Demonstrate awareness of meat safety and quality considerations in practice
40. Clearly convey meat processing ideas in written communication

**Schedule**

(Exact dates subject to change)

| Week of | Lecture Topic | Lab Activity (Thursday) | Reading |
| --- | --- | --- | --- |
| 8/18 | Introduction to ANSC 3700: Meat as a Food | Orientation and safety | 1-6 |
| 8/25 | Structure, Composition, & Development of Animal Tissues | Pork slaughter | 7-53 |
| 9/1 | Muscle Contraction and Energy Metabolism | Pork evaluation & fabrication | 61-74 |
| 9/8 | Conversion of Muscle to Meat (**Exam 1: 9/12)** | Beef slaughter | 97-130 |
| 9/15 | Meat Inspection and Food Safety | Beef carcass evaluation | 291-316 |
| 9/22 | Meat Grading and Evaluation | Beef fabrication - forequarter | 317-349 |
| 9/29 | Meat Merchandising | Beef fabrication - hindquarter | 141-174 |
| 10/6 | Animal Handling (**Exam 2: 10/8)** | Fall break – no lab | 74-96 |
| 10/13 | Properties of Fresh Meat | Field Trip – Golden State Foods | 131-140 |
| 10/20 | Packaging and Storage of Meat | Beef & pork retail cuts and packaging | 235-258 |
| 10/27 | Principles of Meat Processing **(Exam 3: 10/31)** | Beef & pork processing | 175-212 |
| 11/3 | Microbiology of Meat | Beef & pork cookery | 213-234 |
| 11/10 | Palatability and Cookery of Meat | Current issues presentations | 259-275 |
| 11/17 | Nutritive Value of Meat | Iron Chef | 277-290 |
| 11/24 | **NO CLASS – THANKSGIVING BREAK** | Thanksgiving, or practical experience in meat cookery ☺ | --- |
| 12/1 | By-products of the Meat Industry and Food Waste **(Exam 4: 12/5)** | Lab Exam **(12/4)** | 351-363 |
| 12/8 | **Final Exam Period (optional final)**  Final exam date: 12/12, 10:30am – 12:30pm | --- | --- |

**Grading, Assignments, and Class Materials**

**Grading:**

For this course, we will be using **standards based grading**, as opposed to more traditional criterion grading systems. Students will be assessed on whether they have achieved mastery in the learning objectives (LOs) listed above. These assessments will be made based on student responses in exams, assignments, in-class activities, and laboratory participation. For each assessment, the particular objectives being assessed will be clearly designated. Following the assessment, students will have the opportunity to learn from mistakes and re-test on any of the objectives they have not achieved mastery in. The objectives will be assessed in the following ways:

- **Midterm Exams**: There will be **four midterm exams** given during the semester, each testing 8-10 LOs. The knowledge necessary to address the questions will be taught during lecture, laboratory, and from the reading material. These will include multiple choice, fill-in-the-blank, matching, short answer, and essay-type questions. **Exam dates**: September 13, October 9, November 8, December 6.
- **Final Exam**: The optional final exam will be cumulative and include questions that address all LOs. Students will be able to answer questions relevant to objectives they have not yet achieved mastery in. Students will be able to choose which questions to answer and can opt to try for some, all, or none of the LOs. This will not negatively impact course grades
- **Laboratory**: Several LOs will be assessed through the laboratory sections of the class in particular. Material will be presented through recorded lectures posted to Canvas, which students will be expected to watch before their class section. The lecture material will be reviewed at the start of the lab section through a group quiz. Finally, the student’s mastery of the lab-specific LOs (32-36) will be assessed through worksheets, that will be completed during or after the lab section, and through a final lab exam at the end of the semester. Active participation in the labs will also be monitored and used to determine mastery of lab LOs.
- **Current Event Presentations**: To evaluate a student’s ability to understand and discuss current events relevant to meat science, as well as their ability to apply the scientific principles taught in this class to real-world scenarios, students will be evaluated in the form of an extemporaneous oral presentation on a current issue in in the meat industry. The presentations will take place during the lab. This will be assessed using a standardized rubric (see appendix). It will also be used to assess LO 39.
- **Meat Industry Assignment**: This assignment will be used to assess students’ mastery of LOs 38-40 and can also be an opportunity to demonstrate mastery of other objectives if completed correctly. Students will create a business plan for a meat harvest or processing facility using knowledge gained in the course. This will be an individual project. More details are given in the project description in the appendix.

**Assessing Learning Objectives**:

- The instructor and TAs will determine the level of mastery associated with each LO as they are assessed throughout the semester
- Mastery will be assessed on a scale of 0 – 3, with 0 representing no attempt made and 3 representing the expected mastery. A 1 will be assigned if students have attempted to demonstrate knowledge, a 2 will be assigned if students have shown progress towards mastery or partial mastery, and the 3 will be assigned when students fully demonstrate mastery. Please note that some LOs will only be master-able over multiple assessments (for example, LO 34 can only be assessed after both species have been fabricated and by the lab final) so a 2 is the highest possible score for portions of the semester. When this occurs, it will be clarified by the instructors.
- A specific rubric showing what knowledge from the course must be demonstrated for each LO is included in the appendix

| LO Points | Letter Grade |
| --- | --- |
| 102 - 120 | A |
| 84 - 101 | B |
| 66 - 83 | C |
| 50 - 65 | D |
| < 50 | F |

- Student progress toward mastery of each LO will be tracked using the gradebook in Canvas. However, note that the percentage grade given by canvas will not be indicative of the final grade assigned for the course in the traditional method! Students should consider each LO rather than trying to investigate the total grade
- Final grades in the course will be determined by the number of points toward LO mastery the student has achieved (see table)

**Reassessing Learning Objectives**:

- Following each assessment, students will be returned a corrected copy of the exam or assignment. This will indicate how much progress has been made on their understanding of each LO. The LO score will be recorded on the exam and in Canvas at this point.
- Students will then have the opportunity to be reassessed on whichever lecture-based LOs (1-31) they are not satisfied with. This can be done through two pathways:
  1. Re-testing: Students will be given a new set of questions specifically targeted towards the LO they want to work on. These will be administered through the Canvas quiz function but will still be closed note. The number of questions will depend on the level of improvement students wish to demonstrate and the complexity of the LO.
  2. Alternate assessment: Students can demonstrate mastery through a written or creative project. The project must be focused on the knowledge expected in the LO and approved by the instructor before submission.
- There are several requirements students will need to fulfill before being allowed to re-assess:
  - Students cannot retest if they have more than 3 unexcused absences from lecture or more than 1 unexcused absence from lab.
  - Students can reassess **up to 3 LOs per week**, with the exception of the final exam that will give the opportunity to reassess all LOs
  - The procedure to request reassessments is as follows:
  1. The student must engage in an activity that would allow them to improve in their knowledge of the LO. This could include attending office hours, reading the textbook, tutoring, working with the classmate, or other similar activities.
  2.The student will submit their request to reassess in writing to Dr. Belk or a TA during or after lecture on **Monday**. This request should be a sheet of paper describing the activity used to improve knowledge for each LO.
  3. The instructor will then open the quiz on Canvas. Students will have until the following Sunday at 11:59pm to complete this quiz. If the quiz is not completed, it will have to be reopened by the same procedure for another week.
  4. This process can be completed as many times as necessary until a satisfactory score is achieved
  - Note: No mastery level will be reduced by regrading. In other words, a student will not be penalized if they do worse the second time.

**Current Event Presentations**

Through this exercise participants will gain confidence in applying their course material to creatively answering a current issue or “hot topic” in the meat and food industry. It is the goal for this experience that each participant 1) gains more current knowledge of the meat and food industry that they consume daily, and 2) enhance the students ability to think, research and answer a meat and food industry question as a subject expert. This exercise will take place during the assigned laboratory meeting periods. For this exercise, students will utilize previous knowledge of agriculture production systems to aid in their research of the selected topic. Outlined below are the critical thinking expectations of this exercise:

- Select a topic from THE BOWL OF DOOM
- Utilize 5 minutes to research the topic, create your answer outline, and create two illustrations that can aid in your answer.
- Illustrations can be a drawing, power-point slide, image, research data, or any other image that can aid in your answer.
- To complete the exercise, you will have 2 minutes to describe the question and provide an answer to the lab section. 1 minute will be allowed for follow up questions and critique of the answer.
- A summary evaluation following the exercise will be provided with comments to enhance the participants ability to complete this exercise in the future.
- Students will be assessed using the LO 39 rubric to determine the final score.

**Class Materials:**

All materials for this course will be made available on the course Canvas page. Under the “weekly activities” tab there is a page for each week of material. These pages will contain the LOs being covered that week, the lecture slides, the lab lecture recording, any handouts or other materials, and study recommendations. Any other materials that may be helpful to student learning but are not specific to a single week are located under the “more resources” tab. If you are having trouble finding information about the course, please consult this syllabus then both of these Canvas tabs before reaching out to the instructors. Student progress towards LO mastery will be tracked in the Canvas gradebook. Please note again that the final percentage score you will see in Canvas is NOT indicative of your final grade. To track your progress towards your final grade, only look at the “total points” column and compare to the table above.

**Top Hat**:

The Top Hat program administered through Canvas will be used during the lecture portions of this course to take attendance and track understanding via quizzes. Please make sure that you have a Top Hat account. You will be allowed to use a phone, table, or laptop to engage with the Top Hat program during lecture.

**Gradescope:**

Exams will be scanned and graded using the Gradescope tool linked to Canvas. Feedback on performance will be returned to students online using this. If a student has trouble reading or understanding feedback from this source they should contact an instructor.

Appendices

**Current Event Presentation Rubric**

Utilizing the attached definitions below, please complete the rubric for each individual presenting their short answer response to the selected BOWL OF DOOM topic. At the conclusion of the presentations, your scores and comments will be summarized and provided to you for use to improve your speaking skills and if you need to re-assess your LO. Please utilize the results to engage with faculty, graduate students, and your peers to improve on your abilities to address hot topics of the meat industry.

**Date**:

**Name of Presenter**:

**Title or TOPIC:**

**Name of Evaluator**:

1 = Unsatisfactory

2 = Adequate

3 = Average

4 = Good

5 = Excellent

1) Content:	1	2	3	4	5
2) Organization	1	2	3	4	5
3) Language and Delivery	1	2	3	4	5
4) Support	1	2	3	4	5

**What details of this presentation enhanced your knowledge of the topic or Meat and Food Industry?**

**What aspect did the presenter accomplish or do well?**

**Comments for improvement?**

**BUSINESS EXPERIENCE**

This exercise will test your ability to conduct research regarding agriculture production. For this experience you will need to research meat processing (very small to small) plants for drafting your very own meat processing facility. This facility can produce any meat item that you deem would be a **PROFITABLE** production opportunity. Examples can include but are not limited to a boutique butcher shop, jerky production, luncheon meat, custom slaughter and cutting, slaughter facility only, cutting facility only, hot dog or sausage manufacturing to name a few. For completing this exercise, you will need to research and design this facility from either ground up construction, remodeled facility or co-packer. This compiled reported will be uploaded as a PDF document in CANVAS. **DUE 11:59 pm CST November 21, 2025**

**Some example questions that you should consider in your OUTLINE are below. Some or all of these may be important, but it is up to you as a visionary to identify the important information.**

1. Square footage of the facility
2. Number of animals or pounds of product that could be produced each day, week, month
3. Production schedule (52 weeks in a year, are you operating 52 weeks?)
4. Number of employees
5. Federal, State, or Retail Exempt inspection
6. Pricing of production materials (inputs)
   b. Labor
   c. Raw materials
   d. Packaging
   e. Equipment
   f. Sanitation
   g. Electricity
   h. Water
   i. Rendering
   j. Trucking
8. Bank notes (source of funding: Not a WEALTHY relative)
9. Locations of selling
10. Pricing per pound for an estimate of TOTAL revenue
11. Table describing Total Inputs/Expenditures, Total Revenue, Total Profit
12. Schematic/Rendering/Drawing of facility for production
    b. Doors
    c. Windows
    d. Drains
    e. Square footage
    f. Processing Area
    g. Restrooms
    h. Offices
    i. ANYTHING else in a MEAT PRODUCTION Facility

|  | Excellent | Good | Acceptable | Poor | Notes |
| --- | --- | --- | --- | --- | --- |
| Practicality | - Convincing evidence that product is marketable - The facility and production design matches the proposed product - Costs and profits have been analyzed and demonstrate a profitable business model - Project effectively incorporates class material | - Reasonable evidence that product is marketable - Some of the design matches the proposed product - Costs and profits are described - Project incorporates class material | - Product is probably marketable but little or no evidence is provided - Design does not necessarily match the proposed product - Costs and profits are mentioned - Project incorporates very little class material | - Product is not marketable - Design does not make sense for the proposed product - Costs and profits are not mentioned - Project incorporates no class material |  |
| Physical Design | - Layout, design, and flow of space is complete and clearly described - All necessary spaces and equipment are included - Facility design is reasonable and realistic | - Layout, design, and flow of space is described, but may be missing key aspects or details - Most necessary spaces and equipment are included - Facility design is reasonable | - Layout, design, and/or flow of space is described, but with little or no detail - Some necessary spaces and equipment are included with important considerations missing - Facility design is not entirely reasonable | - Layout, design, and flow of space is missing most or all key details - Key spaces and equipment are missing - Facility design does not make sense |  |
| Production Design | - All key and minor production steps are included and described in sufficient detail, including the function and purpose of production steps - Production steps are logical and follow principles described in the course material - Relevant details described in the syllabus are included | - Most key production steps and some minor steps are included and described in detail - Production steps are logical, though logic and course relevancy are lacking - Most relevant details are included | - The provided production steps are very roughly outlined and major steps are missing - Little logic or explanation is provided for included production steps - Many relevant details are missing | - The production design is poorly thought out and missing most major steps and processes - There is no description for why the production is designed the way it is - The details are extremely limited |  |
| Product Quality | - Extensive thought has been put into creating and maintaining product quality - Features protecting product quality align with principles taught in the course material | - Some thought has been put into creating and maintaining product quality, though it may not be sufficient in reality - Most quality decisions match course material, some may be lacking support | - Generally does not include features that will protect or maintain product quality - Quality decisions are only loosely supported by course material | - Product quality has not been considered - Production decisions related to quality contradict material taught in the course |  |
| Product Safety | - Extensive thought has been put into creating a safe and wholesome product - Features protecting product safety align with principles taught in the course material | - Some thought has been put into creating a safe product, though it may not be sufficient in reality - Most safety decisions match course material, though some may be lacking support | - Generally does not include features that will create a safe product - Safety decisions are only loosely supported by course material | - Product safety has not been considered - Production decisions related to safety contradict material taught in the course |  |
| Presentation | - Grammar, spelling, and syntax are near perfect - Structure and organization are logical and easy to follow - Excellent and relevant use of visuals and visual descriptions - Discretionary points for creativity | - Grammar, spelling, and syntax are good with a few large mistakes - Structure and organization are generally logical and easy to follow - Good and relevant use of visuals and visual descriptions | - Grammar, spelling, and syntax errors are distracting - Structure and organization has not been well planned - Some use of visuals and visual descriptions, or use of frivolous visuals | - Grammar, spelling, and syntax errors are poor - No structure or organization - Inappropriate use of visuals |  |

**Supplementary Material 2**

Learning objective rubrics

| **Objective 1.** Describe the role of meat in the US and global economies | | | | | |
| --- | --- | --- | --- | --- | --- |
| **3** | **2** | **1** | | **0** | |
| - Define meat, muscle, and subcategories - Apply meat consumption and production statistics - Describe the role meat and the meat industry has played in the US - Summarize innovations and regulations that have led to the modern meat industry | - Define meat, muscle, and subcategories - Remember meat consumption and production statistics - Recognize the role meat and the meat industry has played in the US - Recognize innovations and regulations that have let to the modern meat industry | - Match meat science terminology with definitions - Select correct consumption and production statistics - Attempt to recognize the role of meat in society | | - Does not meet minimum requirements | |
| **Objective 2.** Illustrate and label the key structural components and myofilaments that make up muscle tissue | | | | | |
| **3** | **2** | | **1** | | **0** |
| - Define the different types of muscle - Diagram or label the organization of muscle, fat, and connective tissue structures - Classify the different proteins within a muscle fiber | - Define most types of muscle - Label most structures in the organization of muscle, fat, and connective tissue - Recognize the different muscle fiber proteins | | - Recognize some muscle types - Recognize some structures in the organization of muscle, fat, and connective tissue - Identify some muscle fiber proteins from a given list | | - Does not meet minimum requirements |
| **Objective 3.** Compare the different muscle fiber types and fatty acids within the livestock animal | | | | | |
| **3** | **2** | | **1** | | **0** |
| - Categorize the different muscle fiber types (I, IIA, IIB) based on biochemical and structural characteristics - Describe the structure of triglycerides and fatty acids - Describe the major fatty acids and triglyceride profiles of livestock animals - Define how muscle fiber and fat types appear in different animals or parts | - Describe most biochemical and structural aspects of muscle fiber types - Recognize the structure of triglycerides and fatty acids - Recognize a few fatty acids and triglycerides | | - Know that there are different muscle fiber types - Select fatty acids from a given list | | - Does not meet minimum requirements |
| **Objective 4.** Describe the process of muscle formation (myogenesis) and growth | | | | | |
| **3** | **2** | | **1** | | **0** |
| - Define the terms associated with tissue formation and structures - Describe the molecular, biochemical, and morphological events that occur during muscle formation - Associate myogenesis activities with muscle function and meat quality | - Define the terms associated with tissue formation and structures - Recognize molecular, biochemical, and morphological events that occur during muscle formation - Draw some connections between myogenesis activities and meat quality | | - Match meat science terminology with definitions - Select some muscle formation events when given a list of terms or descriptions - Acknowledge that there are connections between myogenesis and meat quality | | - Does not meet minimum requirements |
| **Objective 5.** Discuss the necessary conditions for growth and welfare of meat animals | | | | | |
| **3** | **2** | | **1** | | **0** |
| - List the major chemical classes of nutrients - Describe the contributions of each chemical group to normal body functions - Describe procedures used in animal transport and handling from production until harvest - List factors that can contribute to elevated animal stress - Defend the importance of humane handling - Judge the welfare outcomes of different animal handling systems and situations | - List major chemical classes - Connect major chemical groups to given normal body functions - Recognize good and bad live animal handling and transport procedures - Identify some factors that can contribute to animal stress - Know that humane handling is important - Identify major outcomes from different animal handling systems | | - Recognize chemical classes of nutrients from a list - Match some chemical classes with function - Correctly identify some good and bad handling situations - Select from a list factors that might contribute to stress - Know what humane handling is | | - Does not meet minimum requirements |
| **Objective 6.** Describe the process of muscle contraction and relaxation | | | | | |
| **3** | **2** | | **1** | | **0** |
| - List the molecules and ions involved in the action potential and muscle contraction - Summarize the sliding filament theory - Illustrate and label the steps involved in muscle contraction and relaxation - Identify the methods by which muscle fibers generate the energy needed for contraction | - Recognize molecules and ions involved in the action potential and muscle contraction - Fill in points of the sliding filament theory - Label or illustrate the major steps of muscle contraction and relaxation - Recognize sources of energy for contraction | | - Identify from a list some molecules and ions involved in the action potential and contraction - Organize from a list the steps of muscle contraction - Place sources of energy in order of energy contribution | | - Does not meet minimum requirements |
| **Objective 7.** Discuss the metabolic processes necessary for muscle contraction and how this can influence meat quality | | | | | |
| **3** | **2** | | **1** | | **0** |
| - Discuss the relationship between muscle contraction and meat quality - Categorize the biochemical activities involved in ATP generation based on oxygen needs, time, and substrate - Describe where different energy metabolism steps occur | - Recognize the relationship between muscle contraction and meat quality - Correctly categorize some biochemical activities involved in ATP generation - Identify where different energy metabolism steps occur | | - Identify whether or not muscle contraction influences meat quality - Associated biochemical activities with ATP generation - Identify from a list the steps in energy metabolism | | - Does not meet minimum requirements |
| **Objective 8.** Describe the steps and tools used in animal harvest | | | | | |
| **3** | **2** | | **1** | | **0** |
| - Describe the steps of slaughter – names, purpose, and methods - Discuss the food safety, worker safety, and animal welfare considerations that shape the methods used in slaughter - Understand the methods of religious slaughter - Compare differences in slaughter methods across the livestock species | - Describe aspects of the steps of slaughter - Identify points in each step that contribute to safety - Associate activities with different methods of religious slaughter - Sort harvest activities based on species | | - Place steps of slaughter mostly in order - Identify unsafe situations - Match methods of religious slaughter with activities - Identify from a list species-specific harvest activities | | - Does not meet minimum requirements |
| **Objective 9.** Explain the role of meat inspection in the production of safe food | | | | | |
| **3** | **2** | | **1** | | **0** |
| - Recall the history and development of meat inspection - Interpret the food safety regulations that govern meat and food inspection - Categorize the food inspection agencies - Describe meat inspection activities | - Recall some aspects of meat inspection history - Identify regulations that impact food inspection - Associate products with the appropriate inspection agency - Identify some meat inspection activities | | - Acknowledge a history of meat inspection - Recognize from a list some meat regulations - Recognize that different agencies inspect different products | | - Does not meet minimum requirements |
| **Objective 10.** Understand the basics of HACCP and preventative food safety | | | | | |
| **3** | **2** | | **1** | | **0** |
| - List the 7 principles of HACCP - Provide examples of different HACCP hazards that may appear in meat production - Identify the most common food allergens - Use a decision tree to identify CCPs - Understand monitoring, record keeping, and verification activities - Compare HACCP final rule, BRC, and FSMA food safety regulations | - List at least 6 principles of HACCP - Know the three types of hazards - Identify some food allergens - Understand the concepts of CCPS, monitoring, record keeping, and verification, though may not be able to apply the understanding - Know that different food types require different plans | | - Recognize HACCP principles from lists or descriptions - Identify types of hazards from a list - Identify HACCP concepts from a list | | - Does not meet minimum requirements |
| **Objective 11.** Diagram the relationship between live animal condition and meat quality | | | | | |
| **3** | **2** | | **1** | | **0** |
| - Determine the influence different body components have on carcass and meat composition - Describe the relationship between antemortem animal condition and postmortem muscle quality - Identify major meat quality features affected by antemortem and postmortem muscle treatment | - Identify connections between body composition and carcass composition - Identify some quality issues that can be connected to postmortem quality - Identify some quality defects caused by antemortem treatment | | - Identify from a list some meat quality issues driven by live animal condition | | - Does not meet minimum requirements |
| **Objective 12.** Outline the processes that occur in the conversion of muscle to meat | | | | | |
| **3** | **2** | | **1** | | **0** |
| - Explain the biochemical processes that occur during muscle to meat conversion - Connect the metabolic processes with consequences in non-living muscle - List the enzymes involved in conversion - Label the changes in different muscle components | - Assign labels to the process of muscle to meat conversion - List most enzymes involved in the conversion - Label most changes in muscle components | | - Recognize steps of muscle to meat conversion from a list - Acknowledge that these changes affect meat quality | | - Does not meet minimum requirements |
| **Objective 13.** Describe the purpose and methods for assessing meat quality and composition | | | | | |
| **3** | **2** | | **1** | | **0** |
| - Describe the different electronic methods of meat quality and body composition assessment: why they are used, what they measure, how they work, and how they are being applied - Describe the purpose of grading - Compare differences between yield and quality grades - Describe factors considered in yield and quality grading | - Describe most of the features of the different electronic methods - Recognize the purpose of grading - Recognize differences between yield and quality grades - List factors considered in yield and quality grading | | - Match some electronic methods with their uses and functions - Identify quality and yield grades - Determine whether certain factors are important in yield and quality grading | | - Does not meet minimum requirements |
| **Objective 14.** Calculate yield and quality grades for beef, pork, and lamb | | | | | |
| **3** | **2** | | **1** | | **0** |
| - Mathematically calculate yield grades for beef when given the formula - Mathematically calculate yield grades for pork and lamb - Identify quality grades for beef and lamb and quality acceptability in pork | - Use the calculations for yield grades correctly but arrive at incorrect answers - Misuse yield grade formulas but arrive at the correct answers - Describe quality factors for beef, pork, and lamb but cannot correctly apply grades | | - Attempt to use formulas to calculate yield grades - Attempt to assign quality grades or acceptability | | - Does not meet minimum requirements |
| **Objective 15.** Identify the market forms of meat products | | | | | |
| **3** | **2** | | **1** | | **0** |
| - Compare primary market forms used for different meat species - Diagram associations between primal and retail cuts - Match anatomical and common terms to describe different bones and muscles associated with meat products - Associate specific products with their IMPs code | - List market forms but cannot identify all of them - List primals for each species but cannot identify them - Match most anatomical and market terms for bones and muscles - Associate most IMPS products with code | | - List some market forms but cannot identify them - List some primals for each species but cannot identify them - Match some anatomical and market terms for bones and muscles - Know what IMPS is | | - Does not meet minimum requirements |
| **Objective 16.** Describe the factors that influence water holding capacity in meat | | | | | |
| **3** | **2** | | **1** | | **0** |
| - List factors that can influence WHC - Describe biochemically why these factors influence WHC - Know the measurements of these factors in normal meat | - List some factors that can influence WHC - Describe loosely why these factors influence WHC - Know some measurements in normal meat | | - Assign features from a given list with WHC outcomes | | - Does not meet minimum requirements |
| **Objective 17.** Describe the factors that influence meat color | | | | | |
| **3** | **2** | | **1** | | **0** |
| - List factors that can influence color - Diagram the meat color triangle - Describe pigment molecules that drive meat color | - List some factors that can influence color - Label the meat color triangle - Compare molecules that drive meat color | | - Recognize the meat color triangle - Select from a given list molecules and factors that influence color | | - Does not meet minimum requirements |
| **Objective 18.** Identify meat quality defects and recognize the causes | | | | | |
| **3** | **2** | | **1** | | **0** |
| - Identify meat quality defects from images and description - Provide a logical explanation with scientific support for why the defect occurred - Suggest realistic resolutions to quality defects | - Identify meat quality defects from image or description - Provide a suggestion for why the defect occurred that may be supported - Suggest any solution | | - Identify meat quality defects when given a list of choices - Provide an unlikely or entirely unsupported suggestion of why defect occurred | | - Does not meet minimum requirements |
| **Objective 19.** Diagram the four primary drivers of meat spoilage | | | | | |
| **3** | **2** | | **1** | | **0** |
| - Define meat spoilage - List the four drivers of meat spoilage - Diagram the intersections between different spoilage drivers | - Recognize definition of meat spoilage - Recognize the four drivers of meat spoilage - Label intersections between drivers | | - Identify some spoilage markers and drivers from a list - Attempt to show relationships between drivers | | - Does not meet minimum requirements |
| **Objective 20.** Define the difference between food safety and food spoilage | | | | | |
| **3** | **2** | | **1** | | **0** |
| - Fully describe the difference between safety and quality | - Can partially describe the difference | | - Knows there is a difference but cannot explain | | - Does not meet minimum requirements |
| **Objective 21.** Diagram the process of lipid oxidation | | | | | |
| **3** | **2** | | **1** | | **0** |
| - Can outline and describe the steps of lipid oxidation - Describe the impacts of lipid oxidation on meat quality | - List the steps of lipid oxidation - Understand the impact on meat quality | | - Recognize the steps of lipid oxidation when given a list | | - Does not meet minimum requirements |
| **Objective 22.** Compare the advantages and disadvantages of different packaging methods | | | | | |
| **3** | **2** | | **1** | | **0** |
| - Describe different packaging types - List the gasses used in MAP packaging - Compare advantages and disadvantages of packaging methods - Identify packaging types | - List packaging types - Associate given advantages and disadvantages with their packaging method - Recognize packaging types | | - List some packaging methods - Associate some advantages and disadvantages with their packaging method | | - Does not meet minimum requirements |
| **Objective 23.** Describe refrigeration and freezing practices for meat preservation | | | | | |
| 3 | 2 | | 1 | | 0 |
| - Outline different refrigeration and freezing practices - Compare the quality advantages and disadvantages associated with refrigeration and freezing - Describe mechanisms through which cold storage methods preserve meat products | - Understand the difference between refrigeration and freezing - Recognize mechanisms used to freeze meat products | | - Identify definitions of refrigeration and freezing | | - Does not meet minimum requirements |
| **Objective 24.** Describe the use of thermal processing for meat preservation | | | | | |
| **3** | **2** | | **1** | | **0** |
| - Describe thermal preservation methods including dehydration and irradiation - Describe the factors involved in smoking meat products - Identify the different components of smoke | - Recognize thermal preservation methods - Describe most factors involved in meat smoking | | - Identify thermal preservation methods from a given list - Recognize smoked meat | | - Does not meet minimum requirements |
| **Objective 25.** Recognize potential sources and routes of microbial contamination in meat and food products | | | | | |
| 3 | 2 | | 1 | | 0 |
| - Describe methods by which a meat product may become contaminated - Identify locations in meat processing with a higher risk - Explain why different meat products have different risks of contamination | - Recognize some methods by which meat may become contaminated - Identify some locations with a higher risk - Recognize that different meat products have different risks | | - Notice that certain given methods may lead to microbial contamination - When given risk factors, identify which are more concerning | | - Does not meet minimum requirements |

| **Objective 26.** Explain the factors that make meat a good microbial growth medium | | | | | | | |
| --- | --- | --- | --- | --- | --- | --- | --- |
| **3** | | **2** | | **1** | | | **0** |
| - List the intrinsic and extrinsic factors that affect microbial growth - Identify the factors present in meat products - List procedures that can be used to reduce the suitability of meat as a growth medium | | - Recognize intrinsic and extrinsic factors that affect microbial growth - Identify some factors present in meat products - Recognize how some procedures can reduce suitability of meat | | - Define intrinsic and extrinsic factors but maybe cannot list them - Recognize that meat is a good growth medium | | | - Does not meet minimum requirements |
| **Objective 27.** Classify and categorize the primary foodborne microorganisms | | | | | | | |
| **3** | | **2** | | **1** | | | **0** |
| - Describe the different classifications of microbes (i.e., bacteria, virus, etc.) - Categorize common microbes based on intrinsic and extrinsic factor requirements - Classify microbes based on cell type, growth, method of transmission - Recognize the specific microbial species that most commonly cause foodborne illness - Describe the mechanisms of pathogenesis and give examples | | - Classify given microbes by most of the features described in (3) - Recognize most common microorganisms - Recognize the mechanisms of pathogenesis and/or give examples | | - Acknowledge the classifications of microbes - Identify a few common microorganisms - Acknowledge that different microbes cause disease with different mechanisms | | | - Does not meet minimum requirements |
| **Objective 28.** Identify the key factors that are involved in meat palatability and consumer acceptance | | | | | | | |
| **3** | | **2** | | **1** | | | **0** |
| - Describe the factors consumers use to make buying decisions - Describe the 3 components of meat palatability - Describe how producers and processors can influence meat palatability | | - Recognize the factors consumers use to make buying decisions - List the 3 components of meat palatability - Recognize how producers and processors can influence meat palatability | | - Recognize from a given list factors that make up meat palatability | | | - Does not meet minimum requirements |
| **Objective 29.** Discuss the value of meat in the diet | | | | | | | |
| **3** | | **2** | | **1** | | | **0** |
| - Describe the value of meat as a nutritious dietary inclusion (including protein, vitamins, minerals, etc.) - Understand the limitations of meat in the diet - Discuss and debate the current US dietary recommendations for meat consumption | | - Recognize key components of meat nutrition - Recognize key limitations of meat nutrition - Identify what the US dietary recommendations for meat consumption are | | - Identify or list some components of meat nutrition - Identify or list some limitations of meat nutrition - Acknowledge the US dietary recommendations | | | - Does not meet minimum requirements |
| **Objective 30.** Classify the different types of processed meat products | | | | | | | |
| **3** | | **2** | | **1** | | | **0** |
| - Identify different types of processed meat products - Categorize processed meat products - Categorize different types of sausage | | - Identify some types of processed meat products - Categorize some meat and sausage products | | - Acknowledge the different categories of meat product | | | - Does not meet minimum requirements |
| **Objective 31.** Describe the raw materials and ingredients used in meat processing | | | | | | | |
| **3** | **2** | | **1** | | **0** | | |
| - Describe the different methods and ingredients for curing meat - Diagram the formation of a stable emulsion - Evaluate the quality and limitations of different filling and binding ingredients - Understand the consumer perception of different meat processing ingredients - Categorize different meat casing materials based on materials, edibility, strength, and palatability | - Identify the value of cure ingredients - Describe the phases in a stable emulsion - Identify different filling and binding ingredients - Describe the consumer perceptions of processed meats - Identify different meat casing materials from written description | | - List cure ingredients - Define an emulsion - Acknowledge negative consumer perceptions - Acknowledge the different casing materials | | - Does not meet minimum requirements | | |
| **Objective 32.** Describe the meat cure reaction | | | | | | | |
| **3** | **2** | | **1** | | | **0** | |
| - Diagram the cured meat color reaction - Identify the compounds created during meat curing | - Label a cured meat color reaction - Identify some compounds created during curing | | - Recognize components of the cured meat reaction when given a list | | | - Does not meet minimum requirements | |
| **Objective 33.** Identify the uses for edible and inedible meat byproducts | | | | | | | |
| **3** | **2** | | **1** | | | **0** | |
| - Identify the edible by-products originating from livestock animals - Discuss the value of variety meats as a food product - Outline the process of creating valuable products from hides, pelts, and skins - Identify the pharmaceutical byproducts that can be obtained from animal glands and organs | - Identify most specific uses for meat byproducts - Recognize the differences between edible and inedible meat byproducts | | - List some meat byproducts - List some uses for meat byproducts | | | - Does not meet minimum requirements | |
| **Objective 34.** Understand the events that occur during cooking meat products | | | | | | | |
| **3** | **2** | | **1** | | | **0** | |
| - Describe the changes in the biochemical, structural, and sensory traits of meat - List the proper cook temperatures for different products | - Describe some changes that occur in meat during cooking - List some proper cook temperatures | | - Acknowledge that meat changes during cooking - At least identify the temperature danger zone | | | - Does not meet minimum requirements | |
| **Objective 35.** Describe the issue of food waste and methods that can be used to reduce it | | | | | | | |
| **3** | **2** | | **1** | | | **0** | |
| - Understand the systemic and individual causes of food loss and food wasted - Describe and discuss different potential solutions to the problem | - Understand systematic and individual causes of food loss or food waste - Describe some potential solutions to the problem | | - Outline the food waste problem | | | - Does not meet minimum requirements | |
| **Objective 36.** Identify meat processing equipment and meat retail cuts | | | | | | | |
| **3** | **2** | | **1** | | | **0** | |
| - Identify most meat processing equipment used in the course and lab - Identify most basic meat retail cuts by species, primal, name | - Identify some meat processing equipment used in the course and lab - Identify some basic meat retail cuts by species and primal | | - Attempt to identify some meat processing equipment used in the course and lab - Identify the species of meat retail cuts | | | - Does not meet minimum requirements | |
| **Objective 37.** Outline the process of meat fabrication, from carcass to retail cuts | | | | | | | |
| **3** | **2** | | **1** | | | **0** | |
| - Can outline and describe the meat fabrication procedures used in lab | - Can outline or describe most meat fabrication procedures used in lab | | - Can remember some meat fabrication procedures used in lab | | | - Does not meet minimum requirements | |
| **Objective 38.** Demonstrate the correct use of meat lab PPE and knife safety | | | | | | | |
| **3** | **2** | | **1** | | | **0** | |
| - Correctly uses PPE and knives throughout the semester | - Correctly uses PPE and knives most of the time | | - Can correctly use PPE and knives when prompted | | | - Does not meet minimum requirements | |
| **Objective 39.** Debate current issues in meat and animal sciences | | | | | | | |
| **3** | **2** | | **1** | | | **0** | |
| - Scores 4-5s on the oral presentation rubric - Engages in discussion on other presentations | - Scores 3-4s on oral presentation rubric - Pays attention to other presentations | | - Scores 2-3s on oral presentation rubric - Attends other presentations | | | - Does not meet minimum requirements | |
| **Objective 40.** Draft a business plan for a meat processing facility | | | | | | | |
| **3** | | **2** | | **1** | | | **0** |
| - Good or Excellent on business plan rubric | | - Acceptable or good on business plan rubric | | - Poor or acceptable on business plan rubric | | | - Does not meet minimum requirements |

**Supplementary Material 3**

Student course survey

**ANSC 3700 Anonymous Survey**

The purpose of this survey is to assess the effectiveness of the Objective Based Grading (OBG) system used in ANSC3700 this semester. This will help us make the course better in future semesters but will also be part of a small research study evaluating whether this method improves student outcomes. The value of your feedback here cannot be overstated. This survey is optional and completely anonymous. It will not be reviewed until after grades are entered at the end of the semester.

For questions 1-7: On a scale from 1 (strongly disagree) to 5 (strongly agree), how does each statement reflect your feelings about the Objective Based Grading system used in this course?

| 1. The grading system and course expectations were adequately described | 1 | 2 | 3 | 4 | 5 |
| --- | --- | --- | --- | --- | --- |
| 2. This grading system improved my learning and retention of the course material | 1 | 2 | 3 | 4 | 5 |
| 3. I expect to receive a higher grade in this course than I would have in traditional grading | 1 | 2 | 3 | 4 | 5 |
| 4. I felt more confident going into exams under this system | 1 | 2 | 3 | 4 | 5 |
| 5. I felt less anxious going into exams under this system | 1 | 2 | 3 | 4 | 5 |
| 6. I received sufficient feedback in this course | 1 | 2 | 3 | 4 | 5 |

7. Do you want to provide further explanation of any of your answers above?
8. Please share any other thoughts, comments, or suggestions about the OBG system used in this course.
